# A Case of Classical Hodgkin’s Lymphoma Presenting With Intractable Pruritus

**DOI:** 10.7759/cureus.19854

**Published:** 2021-11-24

**Authors:** Gregory Benn, Sneha Adidam, Ahmed Ali, Lekidelu Taddesse-Heath

**Affiliations:** 1 Internal Medicine, Howard University Hospital, Washington, USA; 2 Oncology, Howard University Hospital, Washington, USA; 3 Pathology, Howard University Hospital, Washington, USA

**Keywords:** hypercellular bone marrow, metastatic lymphadenopathy, brentuximab vedotin, pruritus, hyper-eosinophilia, hodgkin lymphona

## Abstract

Hodgkin’s Lymphoma presenting with hypereosinophilia and intractable pruritus is a rare entity. Diagnostic and therapeutic decisions remain a challenge in such patients. Here we report a case of a 25-year-old female with a special case of Hodgkin’s lymphoma. A 25-year-old was admitted to our hospital for intractable pruritus and was found to have significant leukocytosis with eosinophilic predominance. The patient underwent a bone marrow biopsy and mediastinal lymph node biopsy, which confirmed classical Hodgkin’s lymphoma. Furthermore, the patient was given six cycles of adriamycin, bleomycin, vincristine, and doxorubicin (ABVD) chemotherapy and initially showed improvement; however, she relapsed within a short period. Post-treatment positron emission tomography (PET) scan showed interval progression of the disease, and so the patient was referred for a clinical trial with immunotherapy and Brentuximab vedotin.

## Introduction

Pruritus may arise as a primary dermatologic condition or a manifestation of a broad array of systemic diseases [1]. Intractable pruritus with unusual hypereosinophilia requires a comprehensive evaluation for an accurate diagnosis. Hodgkin’s lymphoma (HL) is a neoplasm of lymphoid tissue associated with eosinophilia in 15% of cases [2]. The exact mechanism remains unknown; however, there is some knowledge of various mediators such as interleukin 5 (IL-5) and granulocyte-macrophage colony-stimulating factor (GM-CSF) that may be implicated [3,4]. There is an unmet need to study the prognostic factors in such cases as it is understudied. This is a case of an unusual presentation of Hodgkin’s Lymphoma (HL) in a 25-year-old Hispanic female who presented with a three-month history of intractable pruritus and marked eosinophilia. The report aims to sensitize readers of a rare presentation of HL and to discuss the treatment response in this patient. 

## Case presentation

**Figure 1:**
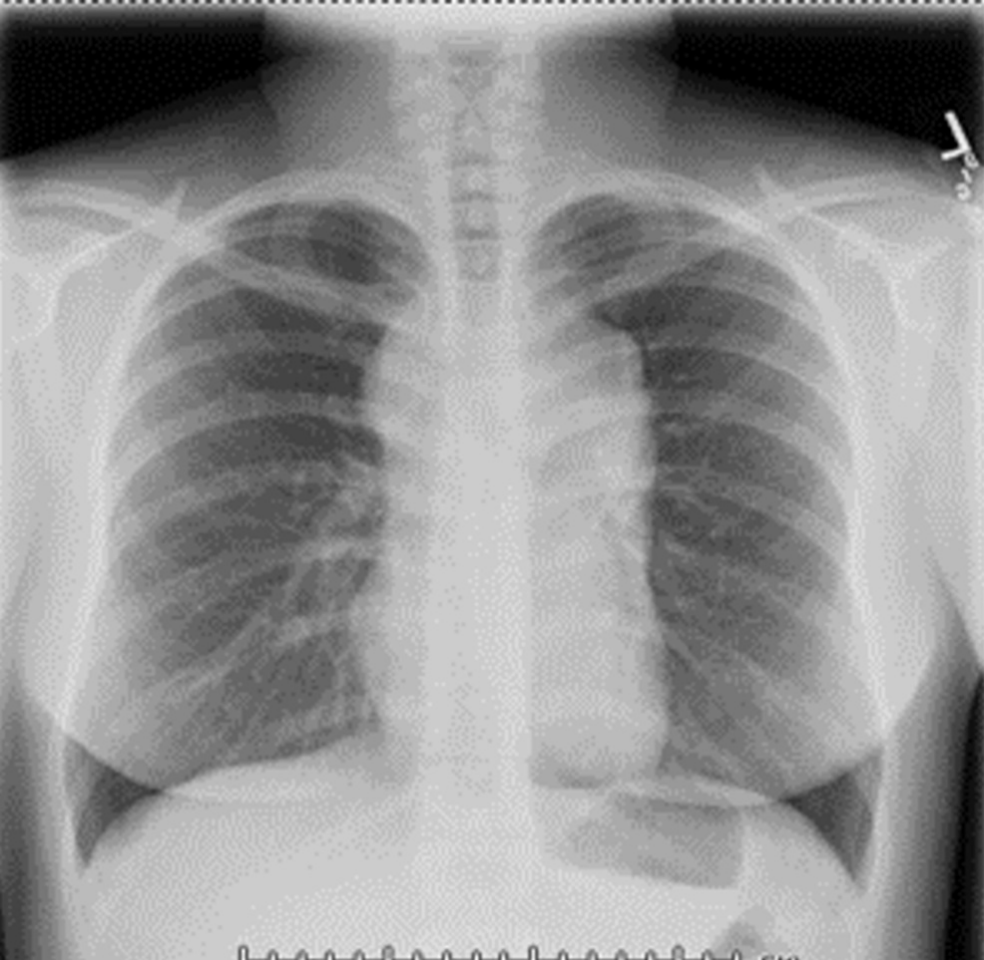
Initial Chest X-ray Revealed Large Mediastinal Adenopathy

**Figure 2:**
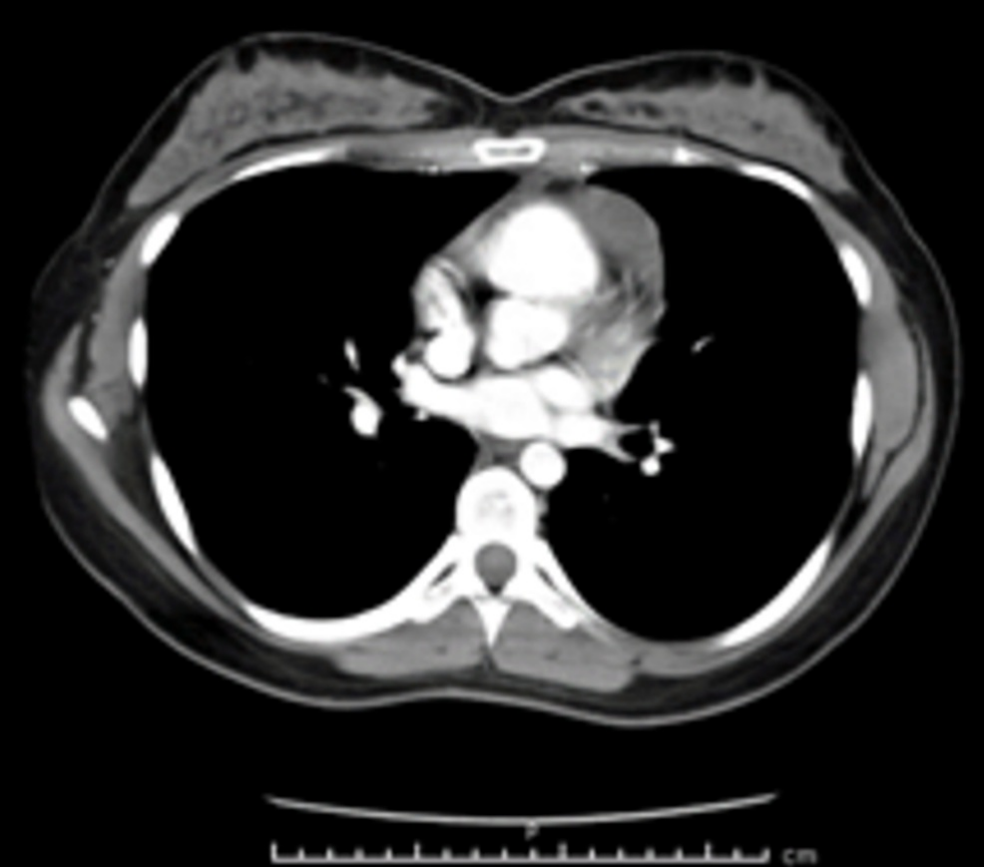
Chest Computed Tomography showing extensive prominent bulky heterogenous lymph nodes in the anterior and middle mediastinum (measures 8 cm in AP dimension and 7 cm transversely), including involvement of the pre-tracheal and perivascular spaces. As a result of the extensive involvement, there is significant narrowing of the left brachiocephalic vein as it traverses the mediastinum. There is also narrowing of the superior vena cava. No clear evidence of hilar adenopathy.

**Figure 3:**
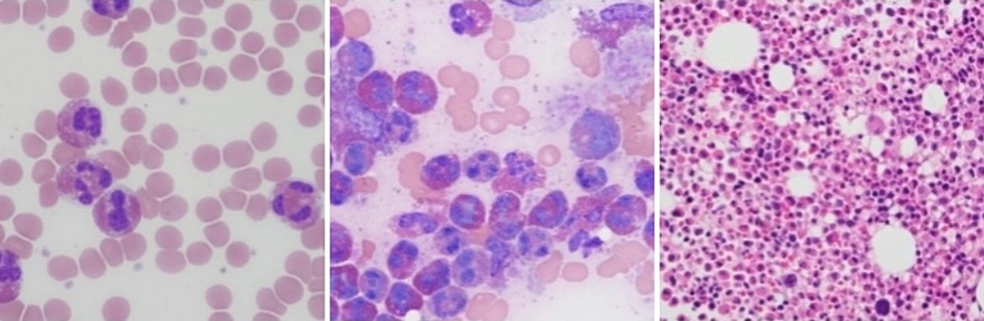
Peripheral smear and bone marrow biopsy. There was a marked increase in eosinophils peripheral smear (left), bone marrow aspirate (middle) and core biopsy (right)

**Figure 4:**
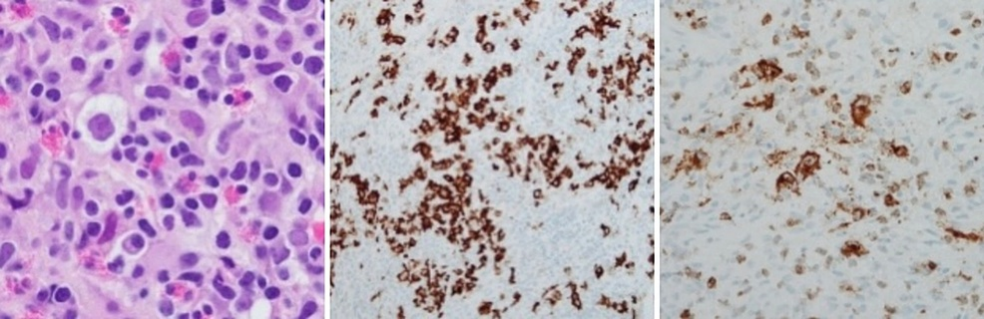
Mediastinal lymph node biopsy. The core biopsy showed marked tissue eosinophilia with scattered large, atypical cells. The large cells had large nuclei and prominent nucleoli. The neoplastic cells were positive for CD 30 and CD 15

This is a case of a 25-year-old Hispanic female who presented with generalized, intractable pruritus for three months. She endorsed areas of purplish discoloration where she had itching and that the itching subsided temporarily in the shower and with topical emollients but worsened with towel drying. Notably, the patient denied any triggers, such as a change in detergents or perfumes. During the outpatient evaluation, she was prescribed hydroxyzine and loratadine. She denied any family history of hematological conditions, including lymphoma/leukemia or urticaria. On arrival, vitals include blood pressure 113/66mmHg, heart rate 126, temperature 98.9 F, respiratory rate 18, and saturation of 100% on room air. Physical examination revealed diffuse petechiae on skin examination, and respiratory examination revealed crackles in the left lower lobe. The rest of the examination was otherwise within normal limits. Laboratory investigations showed that she had a significant leukocytosis of 68.5 x 10^3 with a predominant eosinophilic count of 17.9 x 10^3. The basic metabolic panel was within normal limits. The stool work-up was positive for Dientamoeba Fragilis and was treated with a course of paromomycin. Leukocyte count remained persistently elevated, with an eosinophilic count peaking at 41.93 x10^3. Initial imaging obtained was chest X-ray (Figure 1) which revealed large mediastinal adenopathy. This was followed up with chest computed tomography (CT) (Figure 2), which showed extensive prominent bulky heterogenous lymph nodes in the mediastinum. There was vascular compromise due to extensive involvement of pre-tracheal and perivascular spaces. Further investigations were performed. Bone marrow biopsy (Figure 3) showed mildly hypercellular bone marrow (80%-85%) with marked eosinophilia, decreased iron storage, and negative platelet-derived growth factor receptor (PDGFR). Mediastinal mass biopsy (Figure 4) showed classical HL, CD30+, and CD15+. (Due to a limited sample, it was difficult to subclassify). flow cytometry, fluorescence in situ hybridization (FISH), and cytogenetic of peripheral blood showed no platelet-derived growth factor receptor α (PDGFRA) mutation, no monoclonality, and a normal tryptase level. The PET scan for interim staging showed extensive mediastinal lymphadenopathy, hypermetabolic activity within the lymph nodes standardized uptake value (LN SUV) 4.3-5.8, mass size 5.7cmx4.8cm, another activity noted with enlarged 2.3 cm lymph nodes (LN) adjacent to the cardiac apex, with a comparison with the initial CT it showed a significant reduction in the size of the mass.

**Figure 5:**
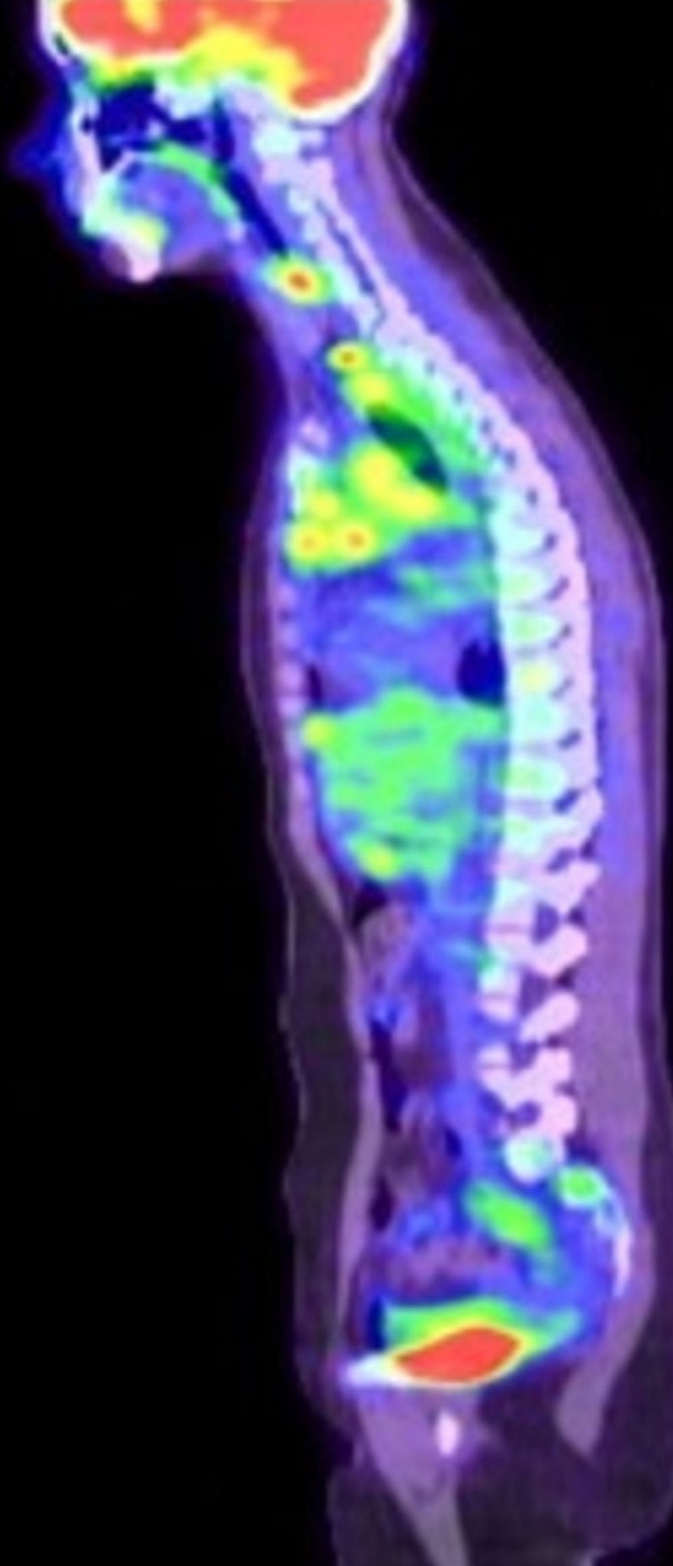
Post treatment PET with disease progression. Neck: shows numerous hypermetabolic lymph nodes. Largest node measures 2.4cm in diameter. Chest: extensive bulky mediastinal adenopathy/soft tissue

**Figure 6:**
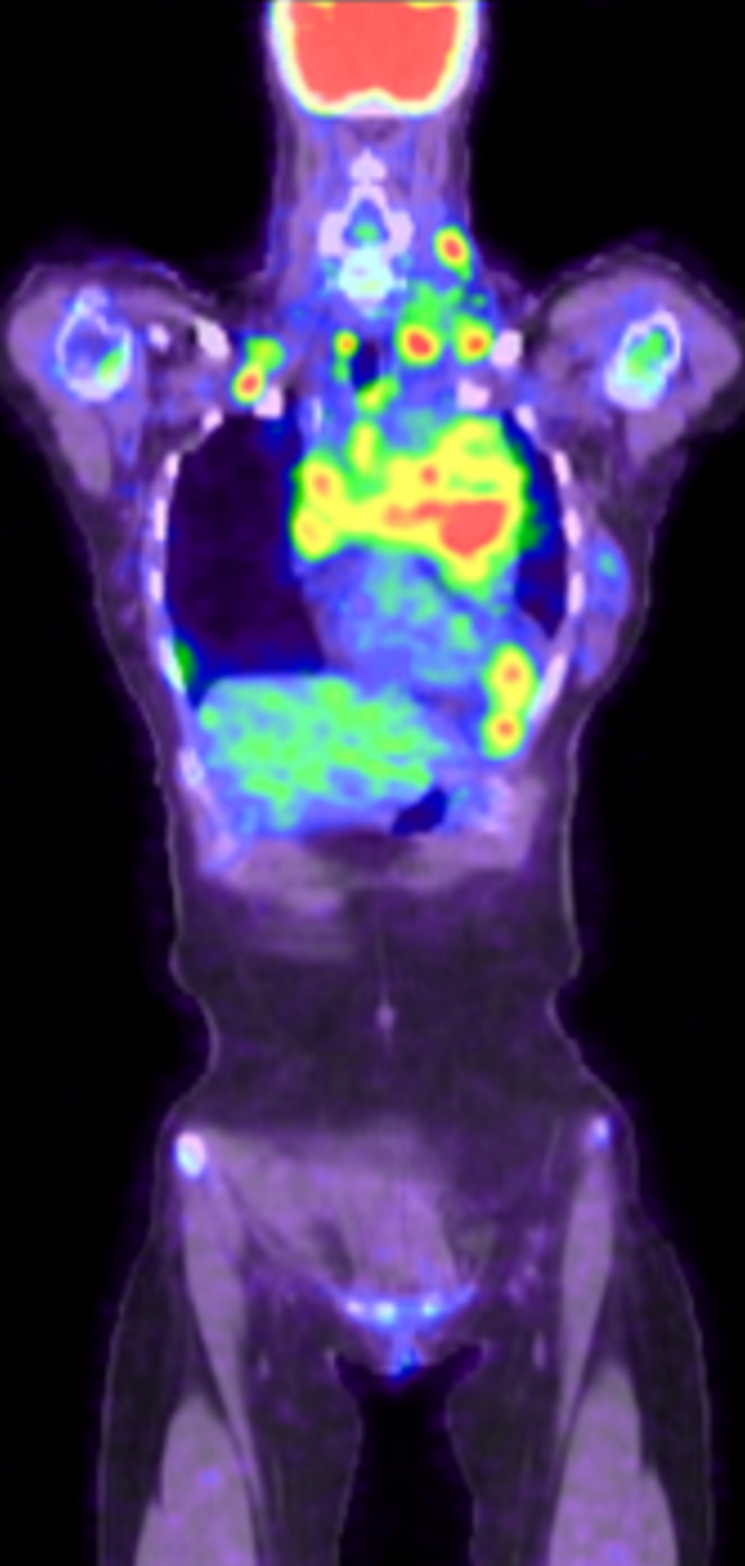
Post treatment PET with disease progression. Neck: shows numerous hypermetabolic lymph nodes. Largest node measures 2.4cm in diameter. Chest: extensive bulky mediastinal adenopathy/soft tissue

She currently follows up in the Hematology clinic and is being managed for classical HL with eosinophilic predominance. She was assigned stage IIA Bulky disease. She received an ABVD regime (adriamycin, bleomycin, vincristine, and doxorubicin) which she tolerated well. Symptomatic treatment (singular, prednisone) for her pruritus was initiated. Gradually, her symptoms progressively improved since the initiation of chemotherapy. Furthermore, her hospital course was complicated by one re-admission for fatigue, tachycardia, and dyspnea, which was later diagnosed as an acute pulmonary embolism on CT pulmonary angiogram and was treated with therapeutic enoxaparin. She was stabilized and discharged with continued follow-up in the hematology clinic. After completion of her six cycles of treatment, she was noted to have normal WBC with normal eosinophil level. However, three weeks after treatment completion, it was noted that her eosinophils level rose again, and her post treatment PET scan (Figures 5, 6) showed interval progression of her disease for which she was referred for a clinical trial that involved the usage of immunotherapy plus brentuximab vedotin.

## Discussion

Upon literature review, the supporting evidence for HL in our index case are as follows: intractable pruritis being the initial symptom, labs that revealed marked leukocytosis with eosinophilia, and a CT chest that showed extensive bulky lymph nodes in the anterior and middle mediastinum with pre-tracheal/vascular involvement. As a result, there was a significant narrowing of the left brachiocephalic vein and superior vena cava.

HL has an incidence of approximately 15% [3] and is characterized by the presence of Reed-Sternberg (RS) cells which are also CD 30 positive in all cases. They are CD15 positive on immunohistochemical staining in 80%-85% of cases. Definitive diagnosis is made by the presence of RS cells and immunophenotyping. Furthermore, primary mediastinal large B-cell lymphoma, anaplastic large cell lymphoma (ALCL), and gray zone lymphoma are some differentials for CD-30 lymphomas. AVD-A (which is brentuximab vedotin, doxorubicin, vinblastine, and dacarbazine) combination has been compared with ABVD combination in a prospective randomized phase III trial [5].

Eosinophilia is an uncommon finding in HL. Thus, there are very few cases reported in the literature [6]. Eosinophilia may be either mild, moderate, or severe based on the number of eosinophils. Hypereosinophilia is defined by two readings of eosinophil level of >1.5 x10^9 with an interval of >1 month between each reading. Furthermore, another entity called hypereosinophilia syndrome is defined by hypereosinophilia and organ damage [7].

Eosinophilia with lymphadenopathy raises the differentials of parasitic infection, especially in certain populations, including immigrants. Furthermore, Gotlib et al. described the identification of the FIP1L1-PDGFRA fusion gene. This may assist in distinguishing chronic eosinophilic leukemia from idiopathic hypereosinophilia. Our index case was negative for PDGFRA [8,9].

The index case was labelled as early-stage IIA bulky disease, which is an unfavorable factor and responded well initially to the ABVD regimen with initial abatement of her symptoms only to recur after six cycles. The standard ABVD regimen usually is associated with a good therapeutic response. However, unfavorable factors and associated hypereosinophilia may have played a role in early relapse. Eosinophils may act as important elements in the pathology of HL by providing cellular ligands for TNF-superfamily receptors (CD40, CD30, CD95/Fas). They can transduce proliferation and antiapoptotic signals at the surface of Hodgkin and Reed/Sternberg (H-RS) cells. Eosinophils may contribute to the disregulated network of interactive signals between H-RS cells, T cells, and other surrounding reactive cells [10].

Due to relapse of symptoms of the disease, our index patient was referred to a clinical trial with brentuximab vedotin and immunotherapy. In a phase II study, brentuximab vedotin was trialed in 102 patients with CD 30 positive HL who relapsed/refractory to autologous stem cell transplant. The overall response rate was found to be 75%, with complete remissions in 34% of patients [11].

## Conclusions

This paper reports the case of a female Hispanic patient in her third decade of life with an atypical presentation of having severe intractable pruritus, skin lesions, hypereosinophilia, and mediastinal adenopathy. The patient quickly relapsed after an initial good response to the standard ABVD regimen for stage II bulky disease. She required treatment with a trial combination of brentuximab vedotin and immunotherapy. Hypereosinophilia, along with bulky disease, may have contributed to her early relapse. This case also serves to provide a rare scenario of eosinophilia and intractable pruritus in HL, which sensitizes physicians to think of HL in similar case presentations in the future. 
